# ﻿A study on the Melandryidae (Coleoptera) of Mount Leigong, Guizhou Province, Southwest China, with descriptions of three new species and a checklist of Chinese melandryid species

**DOI:** 10.3897/zookeys.1261.172411

**Published:** 2025-11-28

**Authors:** Shulin Yang, Hegen Zeng

**Affiliations:** 1 School of Life Sciences, Guizhou Normal University, Universities Town, Huaxi District, Guiyang, Guizhou 550025, China Guizhou Normal University Guiyang China

**Keywords:** Biodiversity, false darkling beetles, taxonomy

## Abstract

Six melandryid species, including three new species, *Phloiotrya similis***sp. nov.**, *Melandrya bifasciata***sp. nov.**, and *Phloeotrinus elongatus***sp. nov.**, and three new provincial records from Guizhou, *Dircaeomorpha elegans* Sasaji, 1974, *Sallumia davidis* Fairmaire, 1889, and *Mikadonius gracilis* Lewis, 1895, from the Leigongshan National Nature Reserve and neighbouring area (Mount Leigong area) are reported in this article. A checklist of Chinese melandryid species is provided.

## ﻿Introduction

Melandryidae Leach, 1815 is a small coleopteran family with approximately 60 genera and 420 species described worldwide (Nikitsky and Pollock 2010). The Chinese fauna of this family is underrepresented. In the “Catalogue of Palaearctic Coleoptera” (Nikitsky and Pollock 2008), 12 species were recorded for China. Based on a synthesis from the works of Konvička (2015a, b) and Nikitsky (2020a, 2020b) in the updated “Catalogue of Palaearctic Coleoptera”, 25 species were recorded in China. Later, with descriptions of one new species of the genus Osphya from Hubei Province (Liu et al. 2023), two new species of Lederina from Yunnan Province (Cosandey 2023a), six new species of Lederina from Taiwan (Cosandey 2023b), one new species of Melandrya from Guizhou (Yang and Liu 2024), one new species of Stenoxylita from Guizhou (Dang and Yang 2024), and the discovery of Perakianus hisamatsui Nakane, 1963 for the Chinese fauna (Yang 2025), along with the earlier record of Dircaeomorpha elegans Sasaji, 1974 in a study on mouthparts of Heteromera (Yu 2005), the number of melandryid species recorded for Chinese fauna increased to 38. Following the resurrection of the family Osphyidae from the subfamily Osphyinae of Melandryidae (Cosandey et al. 2024), three species of the genus Osphya were removed from the Chinese fauna of Melandryidae. The number of melandryid species for the Chinese fauna decreased to 35. We identified 10 melandryid species from specimens collected from the Mount Leigong area and four of these species were treated previously (Dang and Yang 2024; Yang and Liu 2024). Herein, we report on the remaining six species, including three new species, and three new provincial records for Guizhou Province. Currently, 38 species are recorded for the Chinese melandryid fauna.

## ﻿Materials and methods

Specimens collected during the survey in the Leigongshan National Nature Reserve were obtained from crossed panel flight intercept traps (Guizhou Zhongxing Boye Co. Ltd, Guiyang, Guizhou, China) with 99% ethanol as lure (Fig. 4c). Collected specimens were glued on pinned paper cards. Original locality and identification labels, furnished in a combination of handwritten and printed Chinese, are reproduced herein as English translations within double quotation marks. A single forward slash is employed to demarcate line breaks. Red holotype labels and yellow paratype labels were placed beneath the locality labels on type specimens. Morphological characters were examined using an Olympus SZ51 stereomicroscope. Habitus images and type labels were taken with a Canon EOS 5Ds digital camera mounted with a Canon MP-E 65 mm lens. Male genitalia were photographed with the same camera, coupled with an Asahi SMC Takumar 200 mm lens attached to a Mitutoyo M Plan Apo 10×/0.28 objective lens. The camera was mounted on a WeMacro focus rail for capturing images at different focus points. Focus stacking was performed using CombineZP. Specimens are deposited in the School of Life Sciences, Guizhou Normal University, Guiyang, Guizhou, China (**GZNULS**) except other stated.

## ﻿Taxonomy

### ﻿Family Melandryidae

**Subfamily Melandryinae**

**Tribe Dircaeini**

#### ﻿ Dircaeomorpha elegans

Taxon classificationAnimaliaColeopteraMelandryidae

Sasaji, 1974

F9033098-7946-5602-BEC7-584904E5D800

Fig. 1a

##### Material examined.

• “May 24, 2019 / China, Guizhou Province, Leishan County, Queniao Village / Flight Intercept Trap #4 / leg. Shulin Yang”. 26°24.07'N, 108°13.58'E, 1♂, LS19-0125; • “June 30, 2016 / China, Guizhou Province, Leishan County, Queniao Village / Flight Intercept Trap #4 / leg. Shulin Yang”. 26°24.07'N, 108°13.44'E, 1♀, LS16-0510.

**Figure 1.**
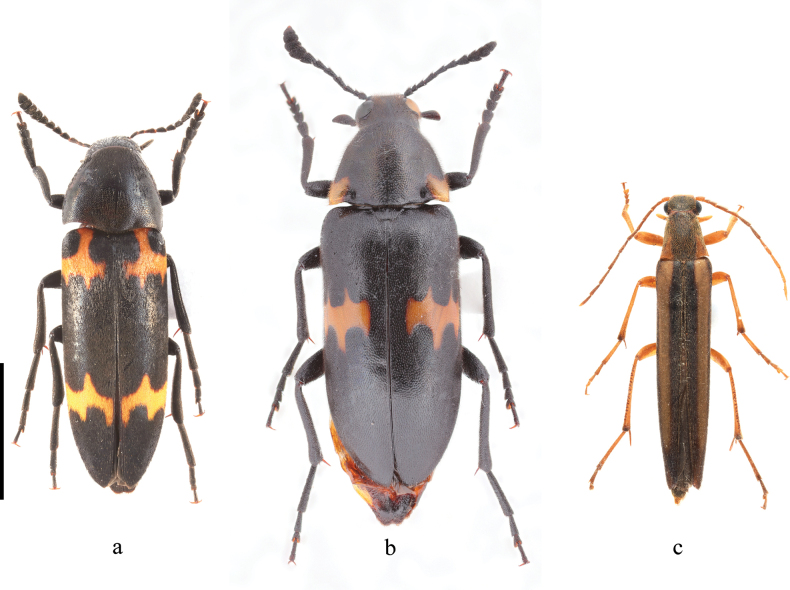
Habitus of Melandryidae species. **a.***Dircaeomorpha elegans* Sasaji, 1974, male; **b.***Sallumia davidis* Fairmaire, 1889, male; **c.***Mikadonius gracilis* Lewis, 1895, male. Scale bar: 5 mm.

#### ﻿ Phloiotrya similis sp. nov.

Taxon classificationAnimaliaColeopteraMelandryidae

954C7D8B-125D-5793-8C8C-356C9B9A2E14

https://zoobank.org/95404FC4-9007-4CF5-B5FE-862E7D551CE6

Fig. 2

##### Material examined.

***Holotype***: • “May 5, 2017 / China, Guizhou Province, Leishan County, Queniao Village / Flight Intercept Trap #1 / leg. Shulin Yang”. 26°24.09'N, 108°13.50'E, 1♂, LS17-0917; ***Paratypes***: • “May 24, 2019 / China, Guizhou Province, County, Queniao Village, Flight Intercept Trap #5 / leg. Shulin Yang”. 26°24.27'N, 108°13.43'E, 1♀, LS19-1562. • “May 24, 2019 / China, Guizhou Province, Leishan County, Queniao Village / Flight Intercept Trap #4 / leg. Shulin Yang”. 26°24.07'N, 108°13.58'E, 1♂, LS19-1010.

##### Diagnosis.

The new species is most similar to its congener, Phloiotrya rugicollis Marseul, 1876 (Fig. 3), the only previously described Phloiotrya species documented with full margined lateral pronotum sides and hair tufts on male abdominal ventrites 1–4 (Nikitsky 1992; Jung 2019). The new species also has hair tufts on male abdominal ventrites 1–4, but it can be distinguished from *P. rugicollis* by the characters of the lateral margins of pronotum and male genitalia. The lateral pronotum margins present only in basal 1/3 of pronotum in *P. similis* sp. nov., while the margins completely extend to anterior angles in *P. rugicollis* (Fig. 3b, c). The median lobe of aedeagus is longer than the parameres, and the parameres are gradually constricted, then apically rounded, in *P. similis* sp. nov., while the length of the median lobe about equals the length of the parameres, and the parameres are abruptly constricted and apically pointed in *P. rugicollis* (illustrated by Jung (2019)). *Phloiotrya similis* sp. nov. differs from other congeners by its densely granulated and slightly depressed, peeling-like middle part of the pronotum, and the dense hair tufts on abdominal ventrites 1–4 in the male.

##### Description.

**Figure 2.**
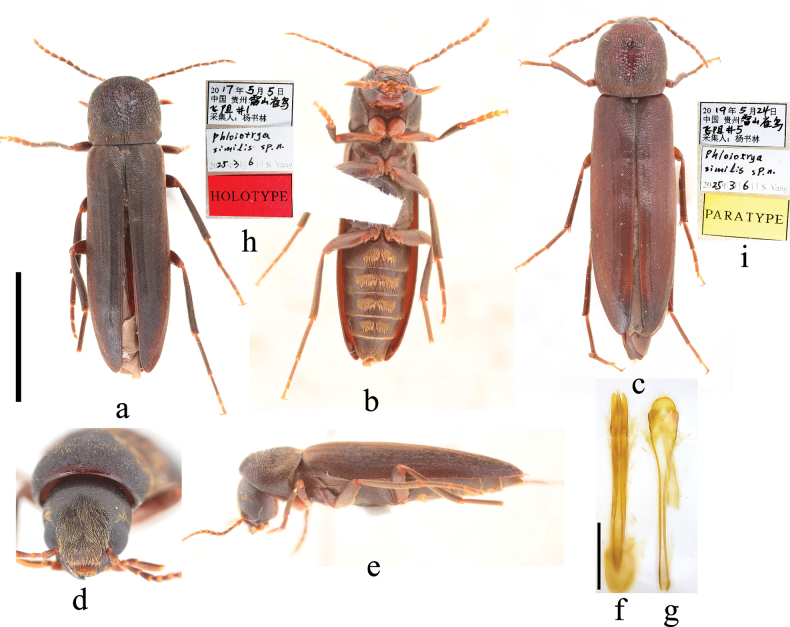
Habitus of *Phloiotrya similis* sp. nov. **a.** Male, dorsal view; **b.** Male, ventral view; **c.** Female, dorsal view; **d.** Male, front view; **e.** Male, lateral view; **f.** Aedeagus, ventral view; **g.** Sternite IX and tergite IX, ventral view; **h.** Holotype labels; **i.** Labels of the female paratype. Scale bars: 5 mm (**a–c**); 0.5 mm (**f, g**); not to scale (**d, e, h, i**).

**Male** (Fig. 2a–e), body oblong; length 10.8–11.7 mm (head concealed under pronotum, and length measured from anterior margin of the pronotum to the apices of elytra), width 2.8–3.0 mm (widest at basal elytra after humeri); black to dark brown, except yellow to yellowish orange on labrum, anteclypeus, procoxae, and basal and ventral sides of profemur; densely punctate, with decumbent, pale yellow setae; setae longer on frons, pronotum, and abdominal ventrites except the hair tufts. ***Head*** (Fig. 2d) about as long as wide, densely punctured, with long, decumbent yellow hairs on frons, anterior of vertex, and posterior of eyes; terminal maxillary palpomere cultriform, longer than other maxillary palpomeres; antenna short, not reaching middle of elytra, brownish black, ratios of antennomeres 1:0.65:1.0:1.0:0.9:0.9:0.85:0.8:0.75:0.75:1.0. ***Thorax*.** Pronotum 2.2–2.7 mm long, widest at basal 1/3, 2.1–2.7 mm, 1.6–2.2 mm at apical margin, nearly parallel-sided, slightly constricted apically and apical 1/4 rounded; with large, irregular granules at middle of pronotum; centre of the granular area slightly depressed and appearing as if peeled off; lateral margins present only in basal 1/3 of pronotum (Fig. 2e); scutellum subquadrate, rounded apically, with dense, pale hairs. Elytra slightly wider than pronotum, parallel-sided at basal 3/4, then gradually tapering towards apices, rounded apically. Legs slender, tarsomeres 1–3 of pro-tarsi slightly widened; prosternal process short and indistinct; mesosternal process short, triangular, pointed apically. ***Abdomen*.** Centre of abdominal ventrite 1–4 with a pair of connected, dense, yellow hair tufts (Fig. 2b). ***Genitalia*** (Fig. 2f, g). Aedeagus elongate; median lobe slightly longer than parameres, gradually tapering and pointed apically; parameres nearly parallel, rounded apically. **Female** (Fig. 2c), length 12.4 mm, width 3.1 mm; generally resembling male but differing from male in lacking hair tufts on abdominal ventrites 1–4 and tarsomeres 1–3 of pro-tarsi not expanded.

**Figure 3.**
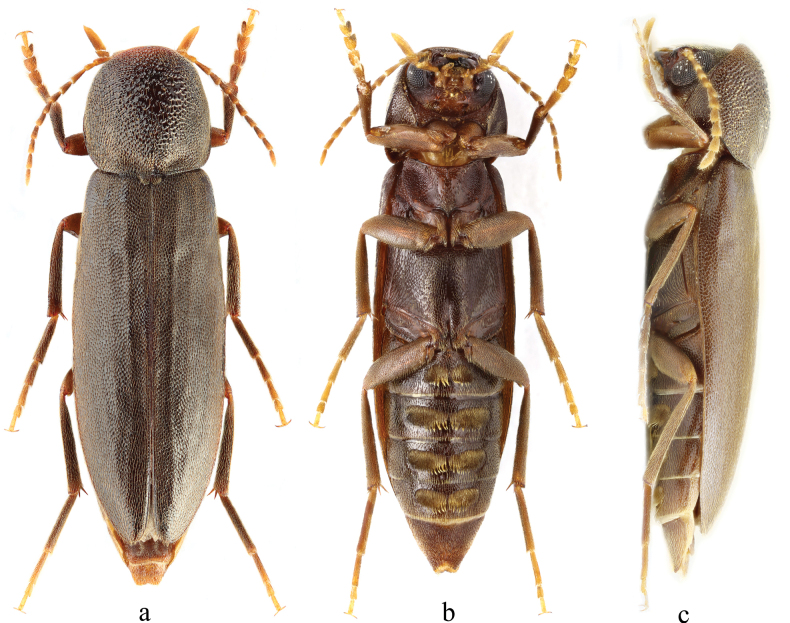
Habitus of *Phloiotrya rugicollis* Marseul, 1876: male. **a.** Dorsal view; **b.** Ventral view; **c.** Lateral view. (Photograph courtesy of Maksim Eduardovich Smirnov, Ivanovo, Russia).

##### Etymology.

The specific name refers to the new species’ similarity to its close congener, *P. rugicollis*. Latin, *similis*, meaning “similar”. An adjective.

##### Collection circumstances.

**Figure 4.**
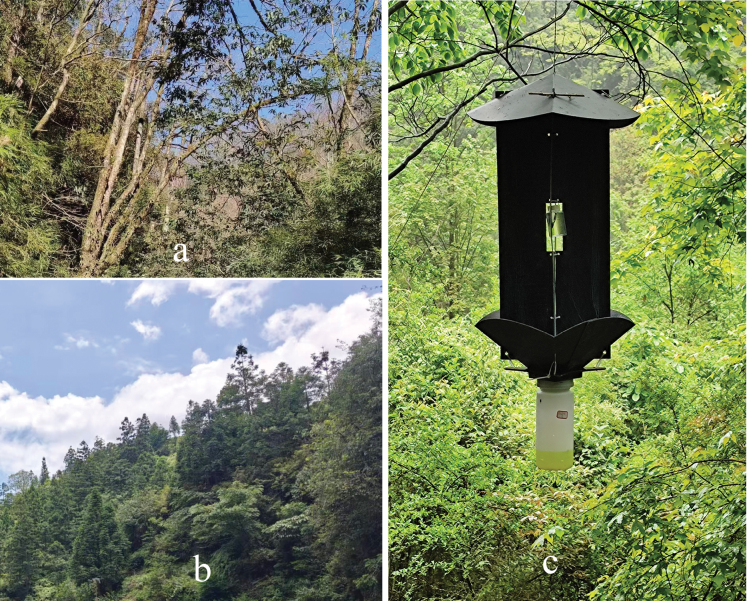
Habitats at collection localities and flight-intercept trap. **a.** Collection locality at Queniao village; **b.** Collection locality at Getou village; **c.** A flight-intercept trap.

Specimens of this new species were collected in an evergreen broadleaf forest of *Quercus multinervis* (W. C. Cheng & T. Hong) J. Q. Li, *Fagus longipetiolata* Seemen and *Fagus lucida* Rehder & E. H. Wilson, near a large tea farm of the village Queniao (Fig. 4a).

##### Distribution.

China: Guizhou Province: Leishan County: Mount Leigong.

Additional material examined.

#### ﻿ Phloiotrya rugicollis

Taxon classificationAnimaliaColeopteraMelandryidae

Marseul, 1876

3BE83F5E-D9E4-59D1-934E-8F6FB699D7C7

##### Note.

Russia, SE Maritime Prov., Lazovsky dist., Lazovsky reserve, ~12 km SW Sokoltchi, attracted to light, June 28, 2010, leg. M. Smirnov. 1♂ (MSCI, Private Collection of Maxim Smirnov, Ivanovo, Russia)

### ﻿Tribe Dircaeini

#### ﻿ Melandrya bifasciata sp. nov.

Taxon classificationAnimaliaColeopteraMelandryidae

5FDE41BC-F7D8-560C-9779-EA9722CD4472

https://zoobank.org/3F1FA916-4D27-4A97-9BB0-A1C722D252F0

Fig. 5

##### Material examined.

***Holotype***: • “May 5, 2019 / China, Guizhou Province, Leishan County, Queniao Village / Flight Intercept Trap #14 / leg. Shulin Yang”. 26°23.50'N, 108°14.20'E, 1♂, LS19-0208; ***Paratypes***: • “June 3, 2016 / China, Guizhou Province, Leishan County, Getou Village / Flight Intercept Trap #2 / leg. Shulin Yang”. 26°23.46'N, 108°14.12'E, 1♀, LS16-1528; • “May 5, 2017 / China, Guizhou Province, Leishan County, Getou Village / Flight Intercept Trap #1 / leg. Shulin Yang”. 26°24.09'N, 108°13.50'E, 1♂, LS17-0897; • “May 15, 2013 / China, Guizhou Province, Leishan County, Wudong Village / Flight Intercept Trap 150#2 / leg. Shulin Yang”. 26°22.10'N, 108°10.20'E, 1♂, LS13-1476; • “May 5, 2017 / China, Guizhou Province, Leishan County, Getou Village / Flight Intercept Trap #2 / leg. Shulin Yang”. 26°23.46'N, 108°14.12'E, 1♀, LS17-0891; • “May 13, 2016 / China, Guizhou Province, Leishan County, Queniao Village / Flight Intercept Trap #3 / leg. Shulin Yang”. 26°24.07'N, 108°13.44'E, 1♀, LS16-1511.

##### Diagnosis.

*Melandrya bifasciata* sp. nov. differs from most of its congeners by the distinct thick bicoloured hairs on its elytra, except for *Melandrya jaromiri* Konvička, 2015. Both species have black and whitish-yellow pubescent elytra and legs. These two species can be distinguished by the general body profile, the shape of pronotum and elytral hair pattern. The body is strongly tapered posteriorly in *M. bifasciata* while it is oblong and moderately tapered posteriorly in *M. jaromiri*. Sides of the pronotum are nearly parallel at basal 1/4–1/3 in *M. bifasciata* while the sides of the pronotum are gradually tapering anteriorly in *M. jaromiri*. Furthermore, the pronotum is not emarginated laterally near basal pronotal sides and lacks humps on the disc in *M. bifasciata*. The pronotum is emarginated laterally near basal pronotal sides and with four humps on the disc in *M. jaromiri*.

##### Description.

**Figure 5.**
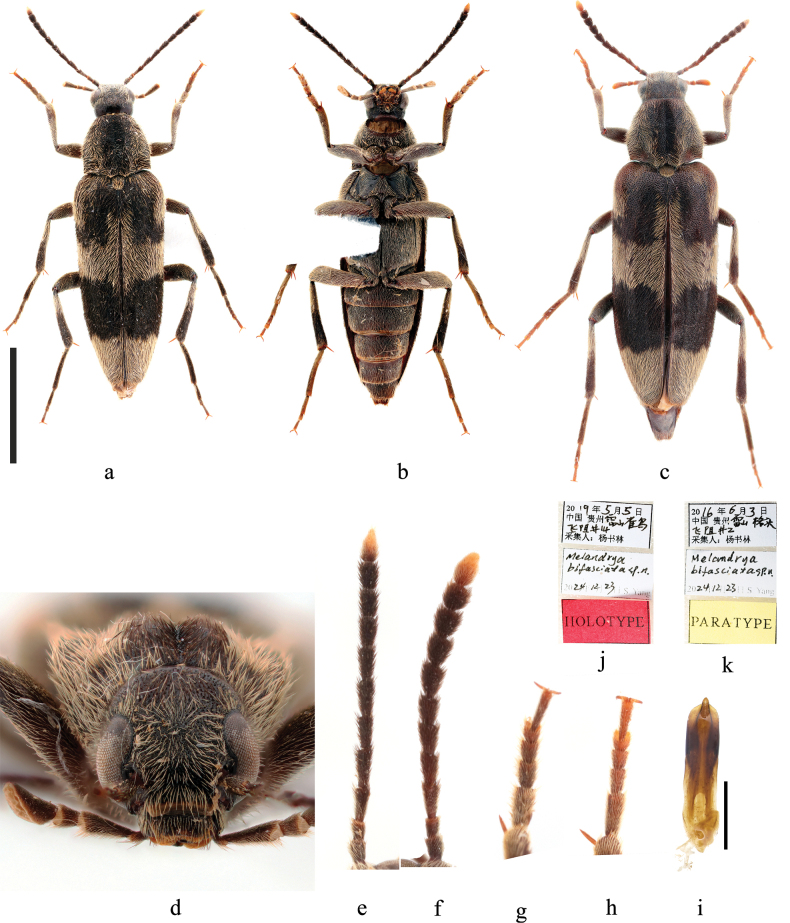
Habitus of *Melandrya bifasciata* sp. nov. **a.** Male, dorsal view; **b.** Male, ventral view; **c.** Female, dorsal view; **d.** Male, front view; **e.** Male antennae, dorsal view; **f.** Female antennae, dorsal view; **g.** Male pro-tarsus, dorsal view; **h.** Female pro-tarsus, dorsal view; **i.** Male aedeagus, ventral view; **j.** Holotype labels; **k.** Labels of a paratype. Scale bars: 5 mm (**a–c**); 0.5 mm (**i**); not to scale (**d–h, j, k**).

**Male** (Fig. 5a, b, d, e, g), body spindle-shaped, length 8.3–10.5 mm (anterior margin of the epistoma to the apices of elytra), width 2.6–3.2 mm (widest at elytra behind the rounded humeri); black, anteclypeus yellow to dark brown; body densely and finely punctate, with decumbent long black and pale hairs. ***Head*** (Fig. 2d) about as long as wide; apex of labrum slightly concave in the middle; terminal maxillary palpomere cultriform, as long as palpomere 2; epistomal suture slightly raised; frons sub-square, with long sparse hairs of an radiation pattern; eyes large, slightly emarginate anteriorly; antenna (Fig. 5e) short, not reaching middle of elytra, black except yellowish orange in apex of antennomere 11; ratios of antennomeres 1:0.7:1.25:1.5:1.2:1.3:1.2:1.15:1:1:1.2. ***Thorax*.** Pronotum 1.7–2.2 mm long, widest at basal 1/3 with width 1.9–2.3 mm, width 1.3–1.4 mm at apical margin, approximately trapezoidal at apical 2/3, and nearly parallel-sided posteriorly, slightly constricted at base; base slightly emarginate mesally and weakly bisinuate; pronotum with a weak longitudinal groove in the middle and slightly depressed longitudinally near basal margin at the middle of each half; hairs in the middle of pronotum black, about 1/3 of pronotum width; scutellum subquadrate, rounded apically, with dense, long, pale hairs. Elytra black, wider than pronotum at humeri, widest at basal 1/8, then tapering towards apices, rounded apically; pale hairs on each elytron presenting three patches, 1) a narrow, inverted L-shaped patch at humerus, stem of the “L” extending posteriorly about 1/4 of elytral length, 2) a transverse patch at the mid of elytron, width about 1/5 of elytral length, extending anteriorly along suture about its width and slightly extending posteriorly along suture, 3) an apical patch, width also about 1/5 of elytral length. Legs slender, most parts of femur and tibia with dense pale hairs except apical 1/4 of pro-tibia, apical half of meso-tibia, and apical half to apical 3/5 of meta-tibia, tarsomeres 1–3 of pro-tarsi slightly widened (Fig. 5g); prosternal process short and indistinct; mesosternal process short, triangular, pointed apically. ***Abdomen*** (Fig. 2b). Pale hairs on ventrites shorter than hairs on elytra and sparser than those on thoracic sternites. ***Genitalia*** (Fig. 5i). Aedeagus elongate, length about 5× width, median lobe longer than parameres, pointed apically. **Female** (Fig. 5e, f, h), length 10.9–12.4 mm, width 3.6–3.9 mm; generally resembling male, differing from male in larger, stouter, and more parallel-sided body, and shorter antenna and tarsomeres 1–3 of pro-tarsi (Fig. 5h) not expanded as in male.

##### Etymology.

The specific name refers to the two transverse hair bands on the middle and apex of elytra. Latin, *bi* (two) and *fasciata* (banded); an adjective.

##### Collection circumstances.

Specimens of this new species were collected in evergreen broadleaf forests of *Quercus multinervis* (W. C. Cheng & T. Hong) J. Q. Li, *Fagus longipetiolata* Seemen, *Fagus lucida* Rehder & E. H. Wilson, *Pinus massoniana* Lamb., and *Cunninghamia lanceolata* (Lamb.) Hook growing in the villages of Wudong and Getou (Fig. 4b).

##### Distribution.

China: Guizhou Province: Leishan County: Mount Leigong.

#### ﻿ Sallumia davidis

Taxon classificationAnimaliaColeopteraMelandryidae

Fairmaire, 1889

507B79D2-EA2D-55A4-AB2B-F0E2E6384E09

Fig. 1b

##### Material examined.

• “May 5, 2019 / China, Guizhou Province, Leishan County, Queniao Village / Flight Intercept Trap #13 / leg. Shulin Yang”. 26°23.45'N, 108°14.08'E, 1♂, LS19-0258; • “May 12, 2021 / China, Guizhou Province, Leishan County, Queniao Village / Flight Intercept Trap #2 / leg. Shulin Yang”, 26°24.09'N, 108°13.49'E, 1♂, LS21-0740; • same data as for the holotype, 1♀, LS19-0993; • “May 24, 2019 / China, Guizhou Province, Leishan County, Queniao Village / Flight Intercept Trap #3 / leg. Shulin Yang”. 26°24.16'N, 108°13.41'E, 1♀, LS19-1067; • “May 20, 2017 / China, Guizhou Province, Leishan County, Queniao Village / Flight Intercept Trap #4 / leg. Shulin Yang”. 26°24.07'N, 108°14.44'E, 1♀, LS17-0878; • “May 24, 2019 / China, Guizhou Province, Leishan County, Queniao Village / Flight Intercept Trap #6 / leg. Shulin Yang”. 26°23.79'N, 108°13.32'E, 1♀, LS19-1019; • “June 2, 2017 / China, Guizhou Province, Leishan County, Queniao Village / Flight Intercept Trap #3 / leg. Shulin Yang”. 26°24.07'N, 108°13.44'E, 1♀, LS17-0467; • “June 2, 2017 / China, Guizhou Province, Leishan County, Queniao Village / Flight Intercept Trap #4 / leg. Shulin Yang”. 26°24.07'N, 108°14.44'E, 1♀, LS17-0430; • “May 5, 2019 / China, Guizhou Province, Leishan County, Queniao Village / Flight Intercept Trap #12 / leg. Shulin Yang”. 26°23.79'N, 108°14.40'E, 1♀, LS19-0428; • “May 24, 2019 / China, Guizhou Province, Leishan County, Queniao Village / Flight Intercept Trap #13 / leg. Shulin Yang”. 26°23.45'N, 108°14.08'E, 1♀, LS19-0981; • “June 3, 2016 / China, Guizhou Province, Leishan County, Queniao Village / Flight Intercept Trap #2 / leg. Shulin Yang”. 26°24.10'N, 108°13.49'E, 1♀, LS16-0412; • “May 24, 2019 / China, Guizhou Province, Leishan County, Queniao Village / Flight Intercept Trap #7 / leg. Shulin Yang”. 26°23.71'N, 108°13.36'E, 1♀, LS19-0564; • “May 12, 2021 / China, Guizhou Province, Leishan County, Queniao Village / Flight Intercept Trap #2 / leg. Shulin Yang”. 26°24.09'N, 108°13.49'E, 1♀, LS21-0662; • “May 13, 2016 / China, Guizhou Province, Leishan County, Queniao Village / Flight Intercept Trap #2 / leg. Shulin Yang”. 26°24.09'N, 108°13.49'E, 1♀, LS16-0254; • “June 3, 2016 / China, Guizhou Province, Leishan County, Queniao Village / Flight Intercept Trap #2 / leg. Shulin Yang”. 26°24.09'N, 108°13.49'E, 1♀, LS16-1053.

### ﻿Tribe Serropalpini

#### ﻿ Mikadonius gracilis

Taxon classificationAnimaliaColeopteraMelandryidae

Lewis, 1895

C8C5E5E2-9AA4-5288-8987-05B2EA5E5461

Fig. 1c

##### Material examined.

• “May 13, 2016 / China, Guizhou Province, Leishan County, Queniao Village / Flight Intercept Trap #2 / leg. Shulin Yang”. 26°24.08'N, 108°13.48'E, 1♂, LS16-0553.

#### ﻿ Phloeotrinus elongatus sp. nov.

Taxon classificationAnimaliaColeopteraMelandryidae

FC3F0472-1424-5835-8122-4DE70E3B5A15

https://zoobank.org/8573DF8B-CFB7-496B-AADF-4382ED9C3C2F

Fig. 6

##### Material examined.

***Holotype***: • “June 9, 2019 / China, Guizhou Province, Leishan County, Queniao Village / Flight Intercept Trap #6 / leg. Shulin Yang”. 26°23.79'N, 108°13.32'E, 1♂, LS19-0437.

##### Diagnosis.

This new species can be distinguished from congeners by characteristics of head, pronotum and male genitalia. The head of *Phloeotrinus elongatus* sp. nov. is not sulcate longitudinally. Head is sulcate medially in *P. femoralis* Lewis, 1895 and *P. filiformis* (Marseul, 1876). Tibiae are not bicoloured in *P. elongatus* sp. nov., while apexes of tibiae are orange in *P. femoralis*. There is a narrow median furrow on the basal 3/4 of pronotum in *P. elongatus* sp. nov., while the pronotum is not furrowed in *P. minusculus* (Nomura, 1962). Compared to characteristics of male genitalia of *P. femoralis*, *P. filiformis*, and *P. minusculus*, as illustrated by Toyoshima and Ishikawa (2000), the parameres are constricted before the apex in *P. femoralis* and *P. filiformis*, while the parameres are not constricted in *P. elongatus* sp. nov. Although the parameres are also not constricted before the apex in *P. minusculus*, the median lobe is longer than the parameres in *P. minusculus* but subequal in *P. elongatus* sp. nov.

##### Description.

**Figure 6.**
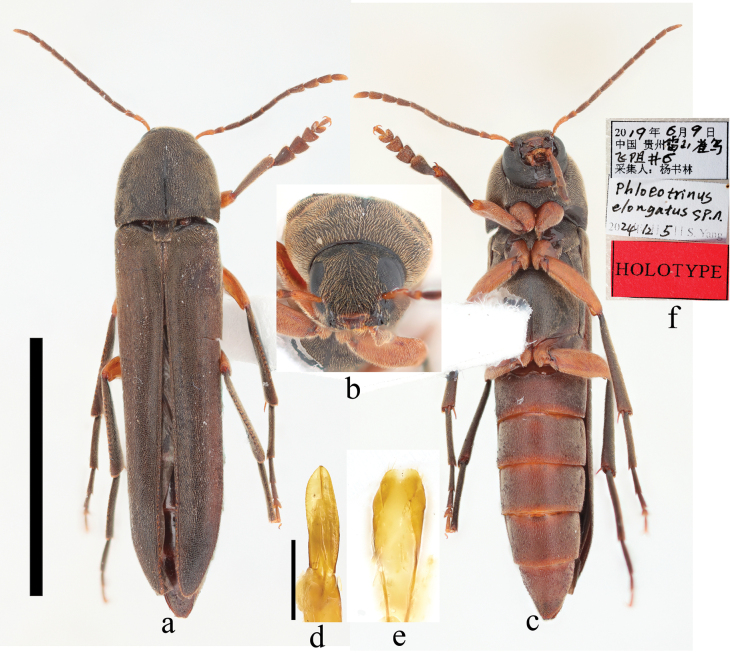
Habitus of *Phloeotrinus elongatus* sp. nov. **a.** Male, dorsal view; **b.** Male, front view; **c.** Male, ventral view; **d.** Male aedeagus, ventral view; **e.** Sternite IX and tergite IX, ventral view; **f.** Holotype labels. Scale bars: 5 mm (**a, c**); 0.5 mm (**d, e**); not to scale (**b, f**).

**Male** (Fig. 6), body elongate, length 9.0 mm (anterior margin of the epistoma to the apices of elytra), width 2.0 mm (widest at elytra after humeri); head, thorax elytra, and tibiae black, maxillary palpi, labrum, anteclypeus, scape, apex of antennomere 2, coxae, femur and abdominal ventrites yellow to dark brown; body densely and finely punctate with pale hairs. ***Head*** (Fig. 6b) about as long as wide; apex of labrum slightly concave in the middle; terminal maxillary palpomere securiform, longer than palpomere 3, about half as long as palpomere 2; frons with dense hairs; eyes large, slightly emarginate anteriorly; antenna short, not reaching middle of elytra, black except scape and apex of antennomere 2, slightly widened apically from antennomeres 3–10; antennomere 11 pointed; ratios of antennomeres 1:0.49:0.79:0.75:0.75:0.81:0.75:0.72:0.67:0.62:0.78. ***Thorax*.** Pronotum 1.8 mm long, widest at base, 2.0 mm, rounded apically; base slightly bisinuate; disc with a weak, longitudinal, glossy groove from base to about apical 1/4; scutellum subquadrate, raised above elytra, with dense hairs on sides. Elytra black, as wide as pronotum at humeri, transversely depressed at base; parallel-sided to apical 1/4, then tapering towards apices, rounded apically. Legs slender, femur yellowish orange; tarsomeres 1–3 of pro-tarsi expanded; tarsomere 4 bilobed underside; outer sides of meso- and meta-tibiae with transverse rows of bristles; prosternal process short (Fig. 6c), widely rounded; mesosternal process long, triangular, rounded apically. ***Abdomen*** (Fig. 6c) dark brown, with dense, pale hairs. ***Genitalia*** (Fig. 6d, e). Parameres not constricted at base, sides parallel, widely tapering and pointed apically. **Female** unknown.

##### Etymology.

The specific name refers to its elongate body. Latin, *elongata* (elongate); an adjective.

##### Collection circumstances.

Same locality and habitat as *P. similis* sp. nov., as given above.

##### Distribution.

China: Guizhou Province: Leishan County: Mount Leigong.

### ﻿Checklist of Chinese Melandryidae

**Subfamily Melandryinae**

**Tribe Dircaeini**

**(1) *Dircaea reducta* Pic, 1954**

**Distribution.** China: Fujian.

**(2) *Dircaeomorpha elegans* Sasaji, 1974**

**Distribution.** China: Guizhou, Yunnan; Japan.

**Remarks.***D. elegans* was first recorded for China from Yunnan Province in a study on mouthparts of Heteromera species (Yu 2005); we cite it here as a distributional record, the specimen referenced is not available for examination.

**(3) *Dircaeomorpha satoi* Ishikawa, Toyoshima & Lee, 2007**

**Distribution.** China: Taiwan.

**(4) *Phloiotrya bellicosa* Lewis, 1895**

**Distribution.** China: Hunnan, Yunnan; Japan; North Korea; Russia; South Korea.

(5) ***Phloiotrya similis* sp. nov. Yang & Zeng, 2025**

**Distribution.** China: Guizhou.

**(6) *Phloiotrya trisignata* Nomura, 1958**

**Distribution.** China: Sichuan; Japan.

**(7) *Stenoxylita quadrifasciata* Dang & Yang, 2024**

**Distribution.** China: Guizhou.

**(8) *Stenoxylita trialbofasciata* Hayashi & Katö, 1956**

**Distribution.** China: Shaanxi; Japan.

**(9) *Wanachia trisignata* Champion, 1916**

**Distribution.** China: Shaanxi; Japan; Russia.

**Tribe Hypulini**

**(10) *Hypulus acutangulus* Lewis, 1895**

**Distribution.** China: Sichuan; Japan.

**(11) *Hypulus cingulatus* Lewis, 1895**

**Distribution.** China: Taiwan.

**Tribe Melandryini**

**(12) *Melandrya bifasciata* sp. nov. Yang & Zeng, 2025**

**Distribution.** China: Guizhou.

**(13) *Melandrya coccinea* (Lewis, 1895)**

**Distribution.** China: Guizhou, Guangxi, Taiwan; Japan; Russia.

**(14) *Melandrya helotoides* Yang & Liu, 2024**

**Distribution.** China: Guizhou.

**(15) *Melandrya incostata* Fairmaire, 1889**

**Distribution.** China: Guizhou, Sichuan, Taiwan; Japan.

**(16) *Melandrya jaromiri* Konvička, 2015**

**Distribution.** China: Hubei.

**(17) *Melandrya minshanensis* Gusakov, 2005**

**Distribution.** China: Sichuan.

**(18) *Melandrya monstrum* Gusakov, 2009**

**Distribution.** China: Yunnan.

**(19) *Phryganophilus ruficollis* Fabricius, 1798**

**Distribution.** China: Sichuan; Japan; Mongolia; Russia; Albania; Austria; Bosnia Herzegovina; Belarus; Croatia; Czech Republic; Estonia; Finland; France; Germany; Latvia; Norway; Portugal; Poland; Romania; Slovakia; Sweden; Spain; Ukraine; Serbia and Montenegro.

**(20) *Sallumia atra* Pic, 1934**

**Distribution.** China.

**(21) *Sallumia davidis* Fairmaire, 1889**

**Distribution.** China: Guizhou, Sichuan.

**Tribe Orchesiini**

**(22) *Lederina armadillo* Cosandey, 2023**

**Distribution.** China: Taiwan.

**(23) *Lederina elongata* Cosandey, 2023**

**Distribution.** China: Yunnan.

**(24) *Lederina formosa* Cosandey, 2023**

**Distribution.** China: Taiwan.

**(25) *Lederina insula* Cosandey, 2023**

**Distribution.** China: Taiwan.

**(26) *Lederina ovata* Cosandey, 2023**

**Distribution.** China: Yunnan.

**(27) *Lederina mozolevskayae* Nikitsky, 2001**

**Distribution.** China: Yunnan.

**(28) *Lederina smetanai* Cosandey, 2023**

**Distribution.** China: Taiwan.

**(29) *Lederina viti* Cosandey, 2023**

**Distribution.** China: Taiwan.

**(30) *Lederina yushanensis* Cosandey, 2023**

**Distribution.** China: Taiwan.

**(31) *Orchesia marseuli* Lewis, 1895**

**Distribution.** China: Fujian; Japan.

**(32) *Orchesia vorontsovi* Nikitsky, 2001**

**Distribution.** China: Yunnan.

**Tribe Serropalpini**

**(33) *Mikadonius gracilis* Lewis, 1895**

**Distribution.** China: Guizhou, Henan, Hunan, Shaanxi, Sichuan; Japan.

**(34) *Mimoserropalpus formosanus* Nomura, 1958**

**Distribution.** China: Taiwan.

**(35) *Perakianus hisamatsui* Nakane, 1963**

**Distribution.** China: Guizhou; Japan.

**(36) *Phloeotrinus elongatus* sp. nov. Yang & Zeng, 2025**

**Distribution.** China: Guizhou.

**(37) *Phloeotrinus minusculus* Nomura, 1962**

**Distribution.** China: Taiwan; Japan.

**(38) *Serropalpus iriei* Toyoshima & Y. Ishikawa, 2000**

**Distribution.** China: Taiwan; Japan.

## ﻿Discussion

To date, only 38 species of Chinese Melandryidae have been recorded, distributed across just 11 provinces of 34 provincial-level divisions in China (province, municipality, autonomous region or special administrative region). Among these, only two provinces, Guizhou and Taiwan, host more than 10 species each. The distribution ranges of many species show substantial gaps between newly recorded localities and their type localities or previously known ranges. For example, *H. acutangulus*, *O. marseuli*, *P. trisignata*, *S. trialbofasciata*, and *P. hisamatsui* were previously known only from Japan before being reported in continental Asia and China (Konvička 2015a; Yang 2025). These findings suggest that the actual species richness of China’s melandryid fauna is likely far greater than documented presently.

## Supplementary Material

XML Treatment for Dircaeomorpha elegans

XML Treatment for Phloiotrya similis

XML Treatment for Phloiotrya rugicollis

XML Treatment for Melandrya bifasciata

XML Treatment for Sallumia davidis

XML Treatment for Mikadonius gracilis

XML Treatment for Phloeotrinus elongatus

## ﻿Additional information

### Use of AI

No use of AI was reported.

### Author ORCIDs

Shulin Yang https://orcid.org/0000-0002-5903-0779

### Data availability

All of the data that support the findings of this study are available in the main text.
